# Anti-lymphoma peptide is inspired by mapping a sequence of four amino acids of KRAI motif as nuclear localization signal of Crlz-1

**DOI:** 10.1016/j.omton.2025.200953

**Published:** 2025-02-20

**Authors:** Joo Hyun Pi, Seung Young Choi, Sung-Kyun Park, Junghyun Lim, Chang Joong Kang

**Affiliations:** 1 Department of Genetics and Biotechnology, College of Life Sciences, Kyung Hee University, 1732 Deogyeong-daero, Giheung, Yongin, Gyeonggi 17104, South Korea; 2 Infectious Disease Research Center, Korea Research Institute of Bioscience and Biotechnology, Daejeon 34141, South Korea; 3 School of Pharmacy and Institute of New Drug Development, Jeonbuk National University, Jeonju 54896, South Korea

**Keywords:** MT: Regular Issue, B cell lymphoma, nuclear localization signal, Crlz-1, Runx/CBFβ, Bcl-6

## Abstract

Peptides of Crlz-1 nuclear localization signal as mapped to be a short KRAI sequence inhibited the proliferation of germinal center-derived Ramos cells from Burkitt’s lymphoma patient. This anti-proliferative effect was mechanistically explained by a cascade of the block of Crlz-1 nuclear movement and consequential failure of CBFβ nuclear mobilization, resulting in the absence of bound Runx/CBFβ heterodimer on the enhancer-promoter of the *Bcl-6* GC master gene. As a consequence of this heterodimer absence, the *Bcl-6* expression was abolished, leading to the down-regulation of *cyclins D1-D3* and the up-regulation of *IRF-4*, *Blimp-1*, and *IgJ* genes. Furthermore, this peptide decreased the production of rRNA in these cells, indicating that the nuclear Crlz-1 as a UTP-3 constituent of ribosomal small subunit processome might be necessary to regulate the biogenesis and/or processing of rRNA, and thereby produce ribosomes necessary for their rapid proliferation. Surprisingly, the KRAI motif peptides had an intrinsic cell-membrane permeability by themselves, and therefore their anti-proliferative and anti-tumor effects were also demonstrated in both the cultured cells and Ramos-xenografted mice just by adding them directly to the culture media or injecting them into tail veins. This definitely paved the prospective road to developing a novel anti-cancer peptide drug against the germinal center-derived B cell lymphoma.

## Graphical abstract

**Figure:**
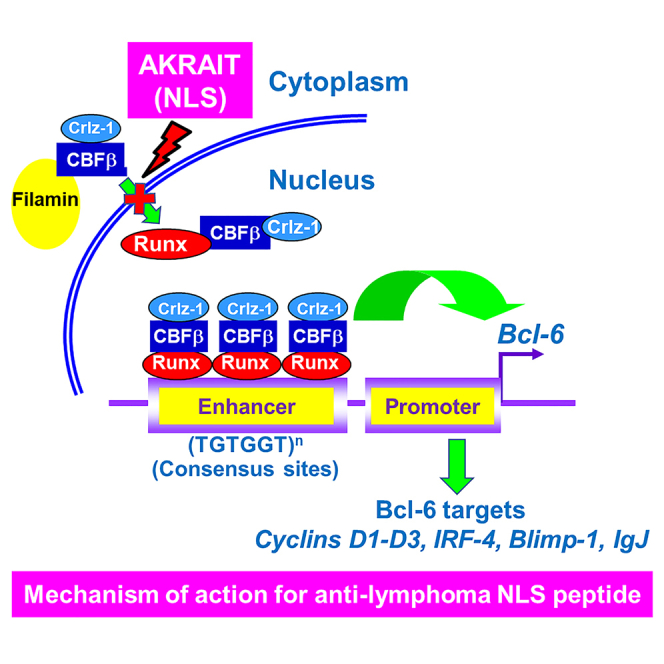

## Teaser

Kang and colleagues report that the blockade of Crlz-1 nuclear movement by AKRAIT as its nuclear localization signal peptide causes the failure of Crlz-1-Runx/CBFβ-Bcl-6 axis cascade and thus the peptide has a significant anti-proliferative and anti-tumor efficacy against the germinal center-derived B cell lymphoma.

## Introduction

Mammalian *Crlz-1* (*charged amino acid-rich leucine zipper 1*) was originally cloned by a yeast two-hybrid method due to its ability to associate with core binding factor β (CBFβ),^1^ and turned out to be the same gene that was specifically expressed in the pre-B and germinal center (GC) centroblast B cells in the vicinity of immunoglobulin J (IgJ) chain gene.^2^^,^^3^^,^^4^ Prior to this cloning of mammalian *Crlz-1*, *Sas10* as a yeast homolog of *Crlz-1* had been cloned in a genetic screen by its protein’s derepressive function.^5^ Recently, Crlz-1 has also been known to play as a UTP-3 (U three protein 3) constituent of ribosomal small subunit (SSU) processome in the nucleolus.^6^^,^^7^

Runx and CBFβ transcription factors are required to heterodimerize in the nucleus to bind to its target DNA regulatory site with a higher affinity.^8^ However, because Runx is a nuclear protein^9^ while CBFβ is a cytoplasmic protein due to its association with filamin A,^10^ a prior nuclear mobilization of cytoplasmic CBFβ must be achieved to allow its hetero-dimerization with Runx. In our previous studies,^11^ Crlz-1 has been shown to be a nuclear protein and to mobilize the cytoplasmic CBFβ into the nucleus to allow this hetero-dimerization of Runx/CBFβ and consequential binding of the heterodimer to its target DNA regulatory sites with a higher affinity.^12^^,^^13^

Interestingly, the promoter of *Crlz-1* gene was found to be bound by LEF-1/β-catenin complex,^3^^,^^14^ indicating that *Crlz-1* would be targeted by the canonical Wnt/β-catenin signaling pathway,^15^ which is known to be involved in the proliferation of many different kinds of cells.^16^^,^^17^ In regard to this linkage of *Crlz-1* expression to the Wnt/β-catenin signaling pathway, Crlz-1 was shown to be important in the rapid proliferative stages of B cell development, especially pre-B cells^3^ and GC centroblast B cells,^2^ where it was shown to be expressed specifically. In pre-B cells, Crlz-1 was found to relay the Wnt/β-catenin signal to the expressions of *cyclins D2-D3* via *VpreB* and *λ5* genes,^3^ while in GC centroblast B cells, Crlz-1 was found to relay the signal to the expressions of *cyclins D1-D3* via *B cell lymphoma-6* (*Bcl-6*) genes.^2^ Notably, it should be noted that many BCLs are quite often generated in the rapid proliferative as well as recombinationally and/or mutationally active stages of B cell development such as pre-B and GC centroblast B cells.^18^^,^^19^^,^^20^^,^^21^ In these contexts, *Crlz-1* has been expected to be a pivotal regulatory point gene in the Wnt/β-catenin signaling network that could be targeted to inhibit the rapid proliferation of pre-B or GC centroblast B cells and their derived lymphomas. As a strategy to regulate or inhibit the function of Crlz-1, its nuclear movement, and thereby its nuclear mobilization of cytoplasmic CBFβ, could be blocked. This strategy was implemented by mapping the nuclear localization signal (NLS) of Crlz-1, the peptide of which might block its nuclear movement by exerting a dominant-negative effect on the interaction between it and an importin-like protein.^22^^,^^23^^,^^24^^,^^25^

## Results

### Mapping of KRAI sequence as the NLS of Crlz-1

**Figure 1:**
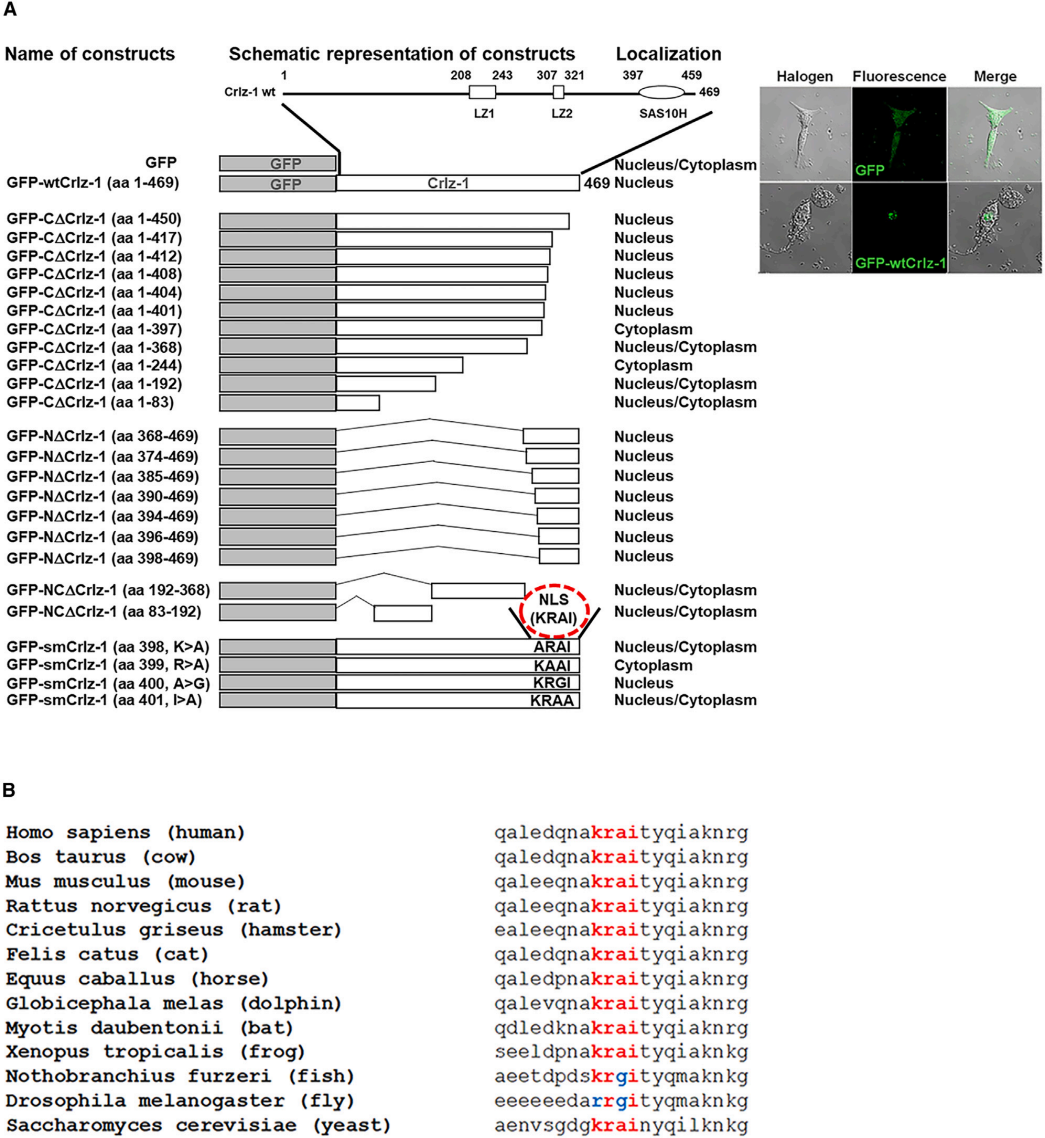
The KRAI sequence is mapped as the nuclear localization signal (NLS) of Crlz-1 (A) The KRAI sequence of four amino acids (aa) from aa 398–401, as indicated by the red dashed circle over the site-directed mutant (sm) constructs at the bottom, turned out to be the NLS of mouse Crlz-1. The various C- and/or N-terminal truncation (CΔ, NΔ, and NCΔ) and sm constructs with their N-terminal ends labeled with GFP are called GFP-CΔCrlz-1, GFP-NΔCrlz-1, GFP-NCΔCrlz-1, and GFP-smCrlz-1 with the remaining or sm amino acids numbered and/or written within their corresponding parentheses. They are schematically drawn in the center, while their corresponding names and cellular locations of expressed proteins are aligned on their left and right sides, respectively. A reference linear view of the full-length (aa 1–469) wild-type (wt) mouse Crlz-1, where the amino acid residues are counted from N to C termini, is drawn at the top and includes two leucine zipper-like domains (LZ1 and LZ2, boxes) and one Sas10 homology domain (Sas10H, oval), as defined by its original cloner. The representative halogen and fluorescent images showing the nuclear localization of GFP-wtCrlz-1 expressed in NIH3T3 cells are given at the top right. (B) The conserved NLS motifs with their surrounding amino acid sequences (right) from 13 species of nearby as well as distant taxonomic lineages are aligned with their corresponding species names (left). As compared to KRAI as the NLS of mouse Crlz-1, the identically or similarly conserved amino acids are colored red or blue, respectively.

A novel NLS of Crlz-1 was mapped by transfecting cells with the various truncation and/or site-directed mutation constructs of mouse *Crlz-1* gene, with their coding sequences linked in-frame to that of green fluorescent protein (GFP), and then examining the cellular localization of their expressed proteins by a fluorescence microscope (Figure 1A). For our purpose of convenient functional sequence and/or domain-mapping analyses, two leucine zipper-like domains (LZ1 and LZ2) and one Sas10 homology domain (Sas10H) as defined originally on the deduced amino acid sequences of mouse Crlz-1 by its original cloner^1^ were again employed as the reference points (Figure 1A, two boxes and one oval as indicated on the linear drawing of the full-length wild-type (wt) mouse Crlz-1 at the top).

As summarized in Figure 1A, the carboxyl (C)-terminally truncated Crlz-1 proteins remained to be localized exclusively in the nucleus until the truncation reached down to amino acid (aa) position 402 as in GFP-CΔCrlz-1 (aa 1–401), as counted from its wt amino (N) terminus. However, a further C-terminally truncated Crlz-1 protein down to aa 398 as in GFP-CΔCrlz-1 (aa 1–397) was found to be localized exclusively in the cytoplasm. Oppositely, when Crlz-1 protein was oppositely truncated up to aa 397 from the N terminus as in GFP-NΔCrlz-1 (aa 398–469), those N-terminally truncated proteins were still exclusively localized in the nucleus. These cellular localization results of both the C-terminally and N-terminally truncated Crlz-1 proteins were collectively interpreted to map the NLS of Crlz-1, which was revealed to the KRAI sequence of four amino acids from aa 398 to aa 401 at the N-terminal tip of the Sas10 homology domain. Among these four amino acids, lysine (K), arginine (R), and isoleucine (I) were found to be essential, but alanine (A) was less critical for the NLS function when we analyzed further the cellular localization of their corresponding site-directed mutant proteins (GFP-smCrlz-1). Other N- and C-terminally truncated Crlz-1 proteins lacking the KRAI sequence as in GFP-NCΔCrlz-1 (aa 192–368) and (aa 83–192) have also lost the exclusive nuclear localization property of wt Crlz-1 protein as expected.

The KRAI sequence as mapped to be the NLS of Crlz-1 has been found to be well conserved among the various species of nearby or quite distant taxonomic lineages (Figure 1B), indicating that the sequence should be critical for the function of Crlz-1 through their evolution. Notably, this NLS sequence also contains the positively charged amino acids such as lysine and arginine, as reported often in the various NLS sequence motifs of other nuclear proteins.^22^^,^^23^^,^^24^^,^^25^^,^^26^ Fortunately, as far as we know, this NLS motif of Crlz-1 consisting of a single stretch of only four amino acids should have not only the unique but also the shortest peptide sequence, and thus should have the inherently potential advantages when the researchers would attempt to develop the peptide as a novel therapeutic drug that might interfere with the relevant intracellular protein-protein interactions by its competitively dominant-negative effect.

### AKRAIT peptide containing KRAI sequence motif as the NLS of Crlz-1 inhibits the proliferation of Ramos cells

**Figure 2:**
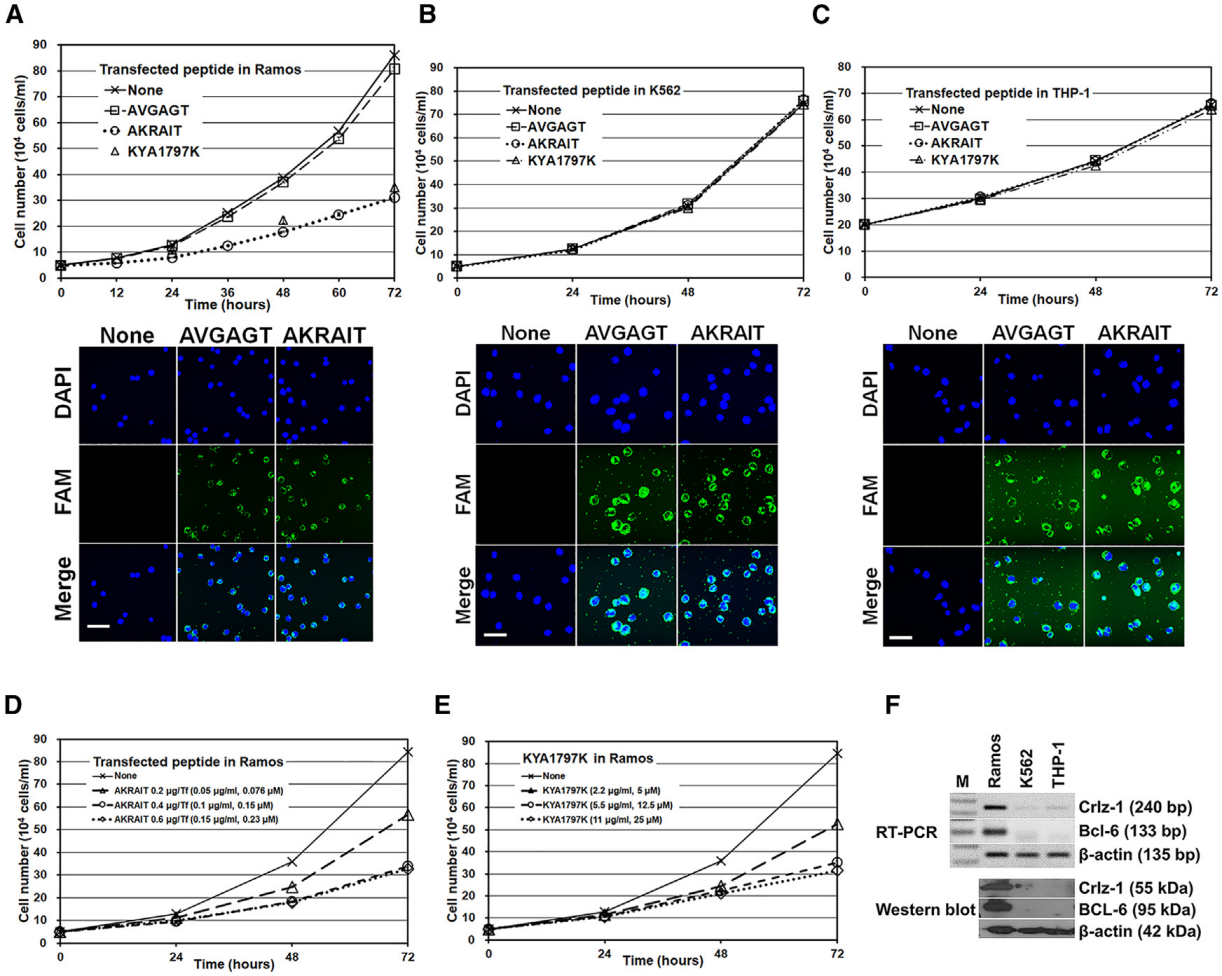
The anti-proliferative effects of AKRAIT peptide (A) The AKRAIT peptide decreased the proliferation of Ramos cells as much as 70% in 3 days after its transfection (Tf) at its saturation dosage (D) of 0.4 μg/Tf as compared to the transfections of PBS (None) and AVGAGT control peptide. The saturation dosage of 0.4 μg peptide per Tf corresponds to the final concentration of 0.1 μg/mL (0.15 μM). KYA1797K, which was employed as the strongest inhibitor of the Wnt/β-catenin signaling pathway to our knowledge, was also used at the saturation concentration (E) of 5.5 μg/mL (12.5 μM). (B and C) The same experiments in the control cells of K562 (B) and THP-1 (C) as performed in (A) did not show any growth-inhibitory effects of the AKRAIT peptide. The confocal fluorescent images, which are shown below each graph of growth curves in (A–C), show the cell membrane permeability of the transfected FAM fluorophore-tagged peptides in the corresponding cells. DAPI is a fluorescent dye for the nucleus. Scale bar: 50 μm. (D and E) Dosage of AKRAIT and KYA1797K versus their cell growth inhibition was titrated to determine their respective saturation concentrations in the absence of any cell death in these Xfect transfections of Ramos cells. (F) The cell lines employed in these experiments were checked for the expression of *Crlz-1* and *Bcl-6* by both reverse-transcriptase PCR (RT-PCR) and western blot experiments, with β-actin as a loading control. The control cells of K562 and THP-1 were found to negligibly express *Crlz-1* and *Bcl-6* genes as compared to Ramos cells. M, size marker of 100-bp DNA ladder. The growth curves with error bars of the standard error of the mean (SEM) were obtained from three independent duplicate transfection experiments. Cells were counted at the indicated time points on a hemocytometer after staining them with an equal volume of 0.4% trypan blue.

**Figure 3:**
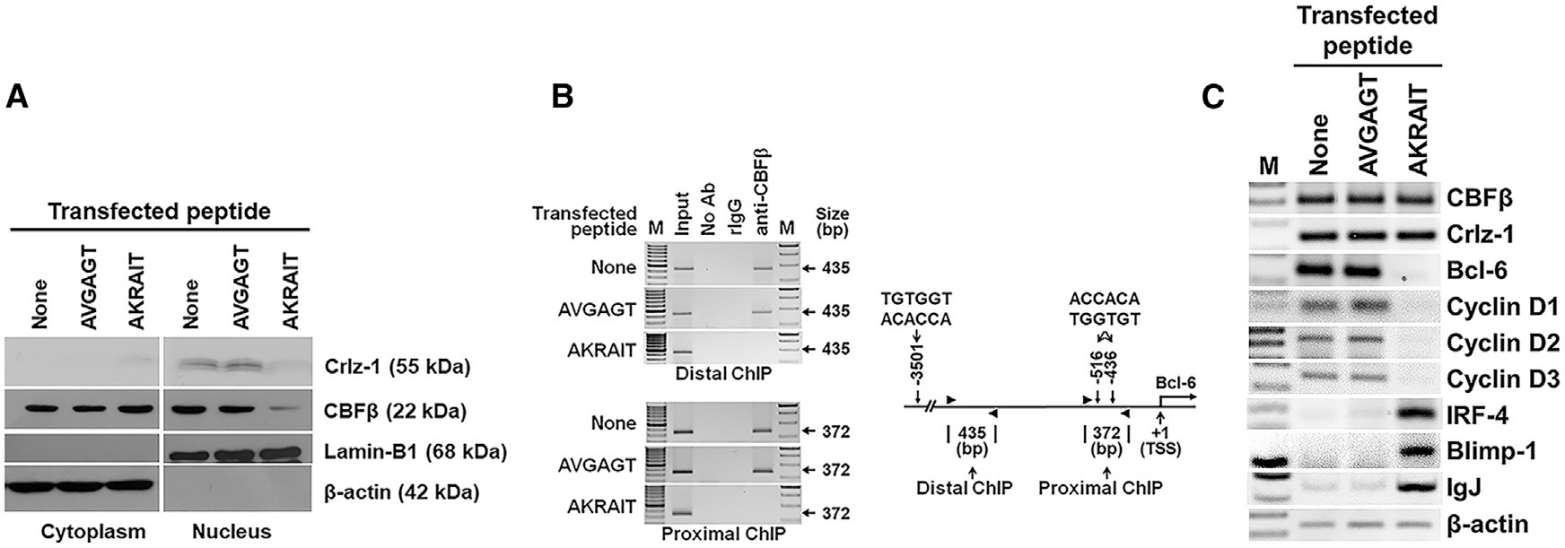
The proliferation inhibitory effect of the AKRAIT peptide is explained by the cellular localization of relevant proteins and its consequential regulation of target gene transcription (A) The AKRAIT peptide blocked the nuclear movement of Crlz-1 with the consequentially failed nuclear mobilization of cytoplasmic CBFβ, which was shown by western blot experiments using the cytoplasmic and nuclear fractions of the peptide-transfected Ramos cells. The name of each relevant protein with its molecular weight (kDa) is aligned on the right side of each blot. (B) The binding of Runx/CBFβ heterodimer on the *Bcl-6* enhancer-promoter region was absent in the AKRAIT peptide-transfected Ramos cells, which was demonstrated by the ChIP experiments. On the right side of the ChIP gel pictures, two PCR amplicons (distal, 435 bp; proximal, 372 bp) on the enhancer-promoter region of *Bcl-6* gene are schematically depicted with the locations (−3501, −516, and −436 from its transcription start site at +1) of Runx/CBFβ consensus (TGTGGT) motifs. Anti-CBFβ, anti-CBFβ antibody; Input, input for ChIP experiment; No Ab, no antibody; rIgG, rabbit IgG control antibody. (C) The transcription levels of various genes associated with the proliferation of GC centroblast cells and their differentiation into plasma cells were analyzed by RT-PCR after harvesting Ramos cells that had been transfected with the corresponding peptides. The transcription of *Bcl-6* was definitely abolished by the AKRAIT transfection, leading to the down-regulation or up-regulation of its various downstream target genes of *cyclins D1*-*D3*, *IRF-4*, *Blimp-1*, and *IgJ*, respectively. The transcription of its upstream *CBFβ* and *Crlz-1* genes in the Crlz-1-Runx/CBFβ-Bcl-6 axis was found not to be affected. AKRAIT, NLS peptide; AVGAGT, mutant control peptide; Lamin-B1 and β-actin, nuclear and cytoplasmic gel loading controls at the level of protein and/or mRNA; M, size marker; None, PBS only.

Initially, to minimize any potentially nonnatural state artifacts of KRAI tetrapeptide fragment because the fragment is not in the natural context of wt protein, we tried to transfect the AKRAIT hexapeptide with additional alanine and threonine residues at the amino and carboxyl sides of the KRAI sequence motif as in the wt Crlz-1 protein into Crlz-1^+^ Ramos cells to see whether it could inhibit their proliferation by its dominant-negative effects on the nuclear movement of Crlz-1. The dominant-negative effects of transfected AKRAIT peptides might be postulated by assuming their competitive inhibition of the potential intracellular interaction of Crlz-1 with an importin-like protein.^22^^,^^25^ Actually, to our great surprise, the transfection of a saturation dosage (Figure 2D) of 0.4 μg AKRAIT peptide into 2 × 10^5^ Ramos cells using the Xfect reagent (experimental procedure detailed in materials and methods) decreased the proliferation of Crlz-1^+^ (Figure 2F) Ramos cells by as much as 70% (Figure 2A) in 3 days as compared to the transfections of PBS (None [i.e., PBS]) and AVGAGT control peptide, where the critical K, R, and I residues of the KRAI sequence motif were replaced by V, G, and G, respectively. However, the transfection of the AKRAIT peptide into K562 (Figure 2B) and THP-1 (Figure 2C) control cells, which are Crlz-1^−^ (Figure 2F), did not show any growth-inhibitory effects at all as compared to the transfections of PBS (None) and the AVGAGT control peptide. Importantly, the peptides were found to be well transfected into the cells in these Xfect transfections, as observed by a confocal fluorescence microscope (Figures 2A–2C, see the fluorescent images below each of the growth curves of the corresponding cells), indicating that the peptides were available for their effects inside the transfected cells. Notably, the death of transfected cells was not observed during this experimental period. This indicates that the decreased cellular proliferation might be due to the arrest of the cell division cycle and/or the final differentiation to plasma cells as supported mechanistically by the decreased expression of proliferation-related *cyclins D1*-*D3* genes, as well as the increased expression of differentiation-related *IRF-4*, *Blimp-1*, and *IgJ* genes, which are shown in Figure 3C. Surprisingly, this proliferation inhibitory effect of AKRAIT peptide in Ramos cells was nearly comparable to the one of KYA1797K,^27^ which is probably the strongest inhibitor of the Wnt/β-catenin signaling pathway to our knowledge,^2^ at their respective saturation dosages in the transfection (0.4 μg/transfection [Tf] AKRAIT) or treatment (5.5 μg/mL KYA1797K) of Ramos cells with the use of Xfect reagent in the absence of any cell death (Figures 2D and 2E).

### The proliferation inhibitory effect of AKRAIT peptide is mechanistically explained by the failed cascade of the Crlz-1-Runx/CBFβ-Bcl-6 axis

The cellular proliferation inhibitory effect of AKRAIT peptide, which was described in the previous section, could be mechanistically explained by the following cascade of phenomena. First in the cascade, the nuclear movement of Crlz-1 was shown to be blocked (Figure 3A), possibly by the dominant-negative effect of AKRAIT peptide on the potential protein-protein interaction between Crlz-1 and an importin-like protein.^22^^,^^23^ The block of Crlz-1 nuclear movement led subsequently to the failure of nuclear mobilization of cytoplasmic CBFβ (Figure 3A). We demonstrated, in accordance with the failure of CBFβ nuclear mobilization, that the binding of the heterodimeric Runx/CBFβ transcription factor on the enhancer-promoter region of *Bcl-6* (the GC master gene)^28^ was absent in our chromatin immunoprecipitation (ChIP) experiments (Figure 3B). Certainly, the transcription of *Bcl-6*, on whose enhancer-promoter region the binding of heterodimeric Runx/CBFβ was demonstrated to be absent, as mentioned above, was truly abolished, ultimately leading to the down-regulation of its proliferation-related target genes such as *cyclins D1*-*D3*,^29^ as well as the up-regulation of its differentiation-related target genes such as *IRF-4*,^30^^,^^31^ *Blimp-1*,^32^ and *IgJ*^4^ (Figure 3C).

### AKRAIT peptide containing the Crlz-1 NLS motif decreases the production of rRNA

**Figure 4:**
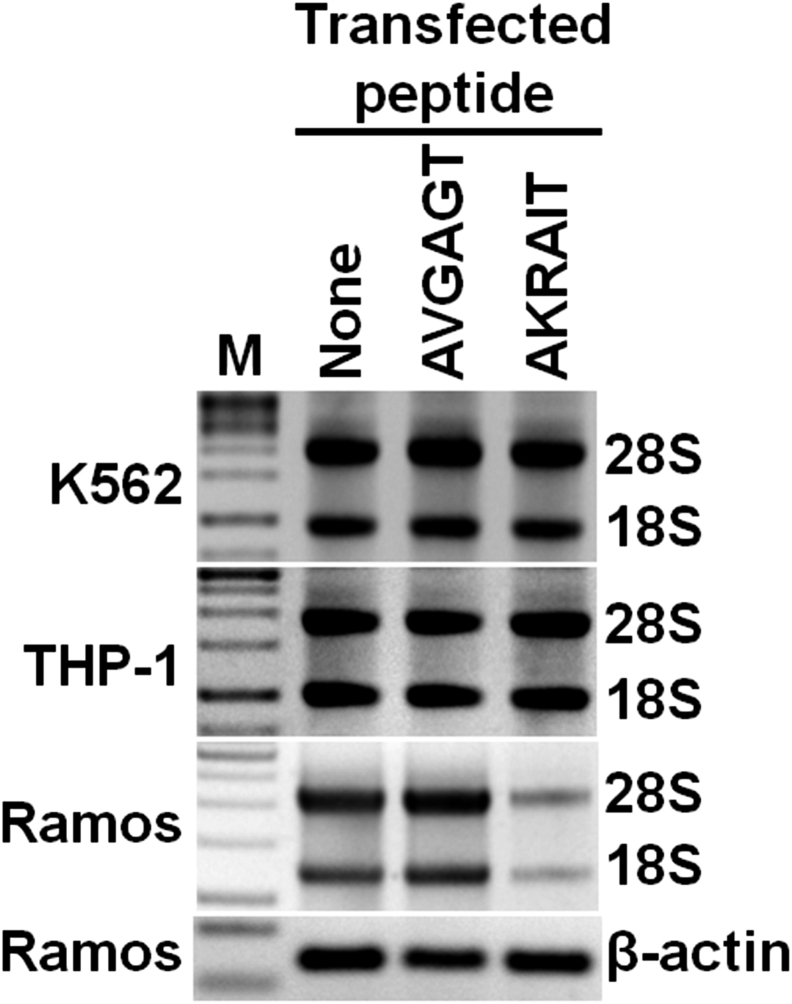
rRNA production is decreased when the AKRAIT peptide is transfected into the Crlz-1^+^ Ramos cells The DNase-treated total RNA, which was normalized by the cell number of each peptide transfection, was loaded on the indicated lane of 1% agarose gel. The two major bands are thought to correspond to 28S and 18S rRNA of the large and small subunits of ribosome, respectively. K562 and THP-1 are Crlz-1^−^ control cells. β-actin, which was amplified by RT-PCR, is also included as a loading control in the case of Ramos cells.

The AKRAIT hexapeptide was also found to inhibit rRNA production in the Crlz-1^+^ Ramos cells but not in the Crlz-1^−^ K562 and THP-1 control cells (Figure 4), possibly indicating that Crlz-1 might also have another nuclear function as a UTP-3 constituent of ribosomal SSU processome^6^^,^^7^^,^^33^^,^^34^ for the biogenesis and/or processing of rRNA in the nucleolus.^6^^,^^7^^,^^33^ This function of Crlz-1 as UTP-3 could be required to regulate the biogenesis and/or processing of rRNA, and thus the production of ribosomes required for the rapid proliferation of GC centroblast-derived Ramos cells. Nevertheless, it has been quite interesting to observe that Crlz-1 is not an essential constituent of rRNA biogenesis and/or processing in all the cells because the Crlz-1^−^ control cells such as K562 and THP-1 proliferate more or less in their own ways as the Crlz-1^+^ Ramos cells do. One possible explanation for this curious observation might be that Crlz-1 as a UTP-3 constituent would be additionally required for the biogenesis and/or processing of rRNA to match up with a very rapid proliferation of pre-B or GC centroblast B cells and their derived lymphomas, but could be substituted by some other factors in different cells as exemplified by a recent report that DCAF13 is required for the rRNA maturation and ribosome biogenesis in the case of rapidly proliferating T cells.^35^ Certainly, an investigation remains to be pursued to clarify this issue in the future.

### The AKRAIT peptide has some intrinsic cell membrane permeability by itself and thus inhibits the proliferation of Crlz-1^+^ Ramos cells when it is added directly to the culture media in the absence of sodium pyruvate

**Figure 5:**
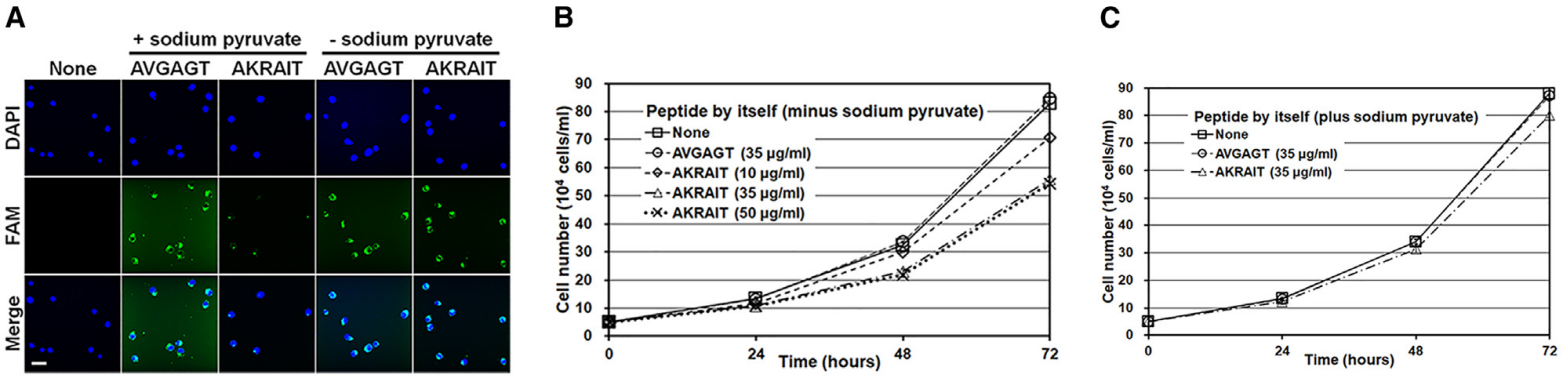
AKRAIT peptide by itself is intrinsically permeable through the cell membrane and thus exerts an inhibitory effect on the cellular proliferation by its direct addition to the culture media in the absence of sodium pyruvate (A) The AKRAIT peptide was shown to be intrinsically membrane permeable in the absence but not in the presence of sodium pyruvate in the cell culture system. The control hydrophobic AVGAGT peptide was shown to be permeable through the membrane regardless of sodium pyruvate. The membrane permeabilities of peptides were observed by their N-terminally conjugated FAM fluorophores. DAPI is a fluorescent dye for the nucleus. Scale bar: 50 μm. (B) A daily addition of 35 μg/mL AKRAIT peptide as compared to the addition of 50 μg/mL was found to be saturated to decrease the proliferation of Ramos cells at about 35% when it was directly added by itself to the cell culture system in the absence of sodium pyruvate. (C) In the presence of sodium pyruvate, the proliferation inhibition by AKRAIT was very slight or negligible as expected from its interference with the membrane permeability of AKRAIT as shown in (A).

Although it was possible to see the dominant-negative effects of the AKRAIT peptide by transfecting it into cells with the aid of Xfect reagent in the cell culture system *in vitro*, this kind of transfection might not be easy to perform in the whole organism system *in vivo*. In the recent literature,^36^^,^^37^^,^^38^^,^^39^ many approaches, including chemical modification and pharmaceutical formulation of peptides, have been suggested to make them permeable through the cell membrane in the cell culture system *in vitro* and eventually in the whole organism system *in vivo*. Among these approaches, the chemical modification of the KRAI motif was first tried in our initial experiments to discover its derivative that could be membrane permeable by itself without the use of Xfect reagent; it would therefore inhibit the proliferation of Crlz-1^+^ Ramos cells in the cell culture system *in vitro* and therefore potentially also the growth of xenografted tumors of Ramos cells in the whole organism system *in vivo*. Significantly, to our great fortune in these preliminary experiments, the initially chosen AKRAIT peptide with the N-terminally conjugated fluorescein amidite (FAM) fluorophore was revealed to have some intrinsic cell membrane permeability by itself (Figure 5A), as in the cases of the well-known cell penetrating peptides (CPPs).^40^^,^^41^ Thereby, the proliferation of Crlz-1^+^ Ramos cells was inhibited by adding it directly to cells in the culture media, which were RPMI-1640 supplemented with fetal bovine serum, l-glutamine, nonessential amino acids, penicillin/streptomycin, and 2-mercaptoethanol, but not sodium pyruvate. Actually, the proliferation of Ramos cells decreased about 35% after 3 days by directly adding a daily saturation amount of 35 μg/mL AKRAIT peptide by itself in 2-mL culture in the absence of sodium pyruvate (Figure 5B). This saturation dosage of 35 μg/mL was determined in a titration experiment where a daily dosage of 50 μg/mL AKRAIT peptide showed a similar inhibition of cellular proliferation (Figure 5B). Sodium pyruvate, which is usually added to the culture media as an additional ingredient to help the growth of cells, has been found to interfere with the membrane permeability of the AKRAIT peptide (Figure 5A; see discussion for a possible explanation for the interference effect of sodium pyruvate on the cell membrane permeability of the AKRAIT peptide). Consistent with the interference effects of sodium pyruvate on the cell membrane permeability of the AKRAIT peptide (Figure 5A), the proliferation of Ramos cells was very slightly or negligibly inhibited by the direct addition of the AKRAIT peptide to the culture media in the presence of sodium pyruvate (Figure 5C).

### Additional control peptides TIARKA and ADEAIT verify further the specific dominant-negative effects of the AKRAIT peptide

**Figure 6:**
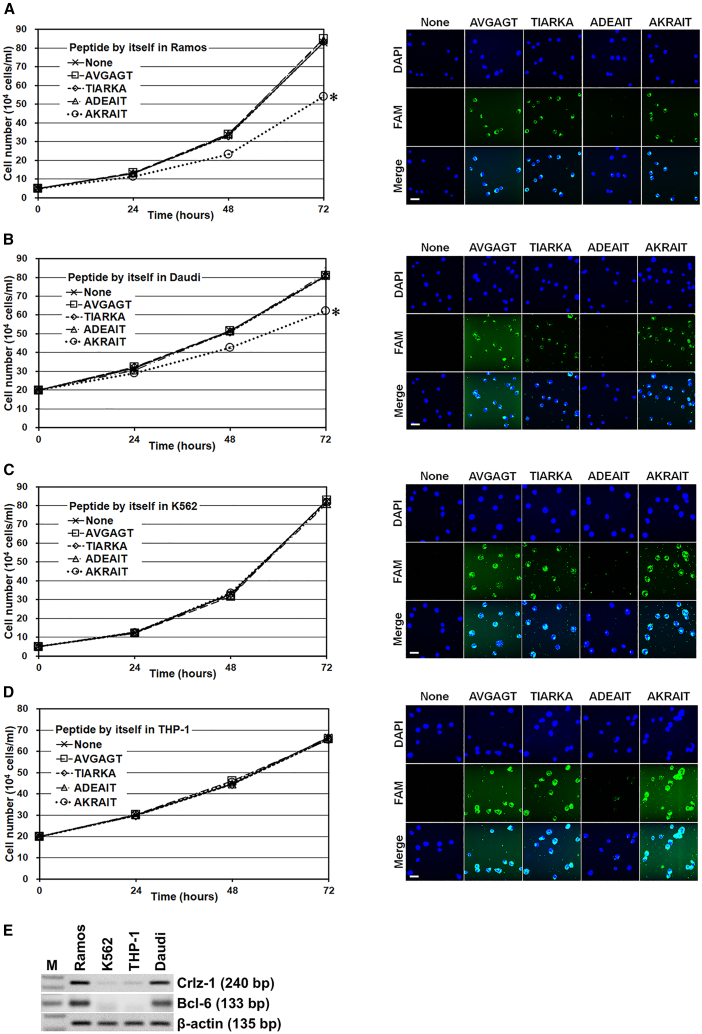
Two additional control peptides verify further that the sequence directionality and positive residues of the AKRAIT peptide are critical for the inhibition of cellular proliferation when it is added directly to cells in the culture media (A and B) The proliferation of Crlz-1^+^ Ramos and Daudi cells was inhibited by AKRAIT but not by three control peptides of hydrophobic AVGAGT, oppositely sequenced TIARKA, and negatively charged ADEAIT. Actually, when the saturation amount of 35 μg/mL (see Figure 5B) of the AKRAIT peptide was added directly to cells daily in the culture media that did not contain sodium pyruvate, the proliferation of the Crlz-1^+^ Ramos (A) and Daudi (B) cells was inhibited by about 35% (∗*p* ≤ 0.0000029, AKRAIT versus None) and 30% (∗*p* ≤ 0.00097, AKRAIT versus None) after 3 days, respectively. (C and D) When the same experiments as in (A) and (B) were performed using Crlz-1^−^ K562 and THP-1 control cells, the specific proliferation inhibitory effects of AKRAIT were not observed. The fluorescent images shown on the right of each corresponding graph of the cell growth curves definitely reveal the intrinsic cell membrane permeabilities of the FAM fluorophore-labeled peptides of AKRAIT, AVGAGT, and TIARKA, but no such intrinsic cell membrane permeability of ADEAIT. Any natural background fluorescence of cells was not detected as shown in the control images of PBS only (None). DAPI is a fluorescent dye for the nucleus. Scale bar: 50 μm. (E) *Crlz-1* and *Bcl-6* were also shown to be expressed in Daudi cells as checked by RT-PCR. The results of Ramos, K562, and THP-1 cells are the same as in Figure 2F. β-Actin is a loading control. M is a size marker of 100-bp DNA ladder.

In addition to the initially chosen control peptide AVGAGT with hydrophobic side chains, two additional control peptides TIARKA and ADEAIT, which were designed by reversing the sequence of AKRAIT or replacing the positively charged critical lysine and arginine residues by the negatively charged aspartic and glutamic acid residues, were intended to be included in these direct peptide addition experiments to verify the values of not only the sequence direction of AKRAIT but also the positively charged lysine and arginine residues for its specific inhibitory function and/or intrinsic cell membrane permeability. Of course, all three control peptides did not show any inhibitory effect on cellular proliferation when they were added directly to four different cells in culture media (Figure 6). The effects of all those peptides, including the inhibitory AKRAIT and the three control peptides on the proliferation of Crlz-1^+^ (Figure 6E) Ramos and Daudi cells (Figures 6A and 6B), as well as the Crlz-1^−^ (Figure 6E) K562 and THP-1 control cells (Figures 6C and 6D), were convincingly supported by checking their cell membrane permeabilities when they, in the forms of N-terminally conjugated FAM fluorophore, were added directly to the cells in culture media (Figure 6, see the fluorescent images at right of growth curves for the corresponding cells). The originally chosen AVGAGT control peptide, which does not inhibit cellular proliferation, was found to pass through the cell membranes possibly by diffusion due to its hydrophobic nature. The oppositely sequenced TIARKA control peptide, which was employed to evaluate the value of sequence directionality of the AKRAIT peptide, was demonstrated not to inhibit cellular proliferation despite its intrinsic cell membrane permeability. The negatively charged ADEAIT control peptide, which was employed to evaluate the critical value of positively charged residues of AKRAIT peptide for its intrinsic cell membrane permeability, was shown to neither pass the cell membrane nor inhibit the cellular proliferation as expected. The experiments performed using all these inhibitory and control peptides indicated that the sequence direction and the positively charged lysine and arginine residues of the AKRAIT peptide are essential for its specific proliferation inhibitory function and its intrinsic cell membrane permeability in the Crlz-1^+^ Ramos and Daudi cells, although its intrinsic cell membrane permeability was also seen without any inhibitory function in the other two Crlz-1^−^ K562 and THP-1 control cells. Finally, the PBS (None) control experiments were included not only to show the normal proliferation of four different cells without any addition of peptides but also to demonstrate that the cells did not have any natural background fluorescence.

### The AKRAIT peptide inhibits the growth of Ramos-xenografted tumors in the athymic nude mice

**Figure 7:**
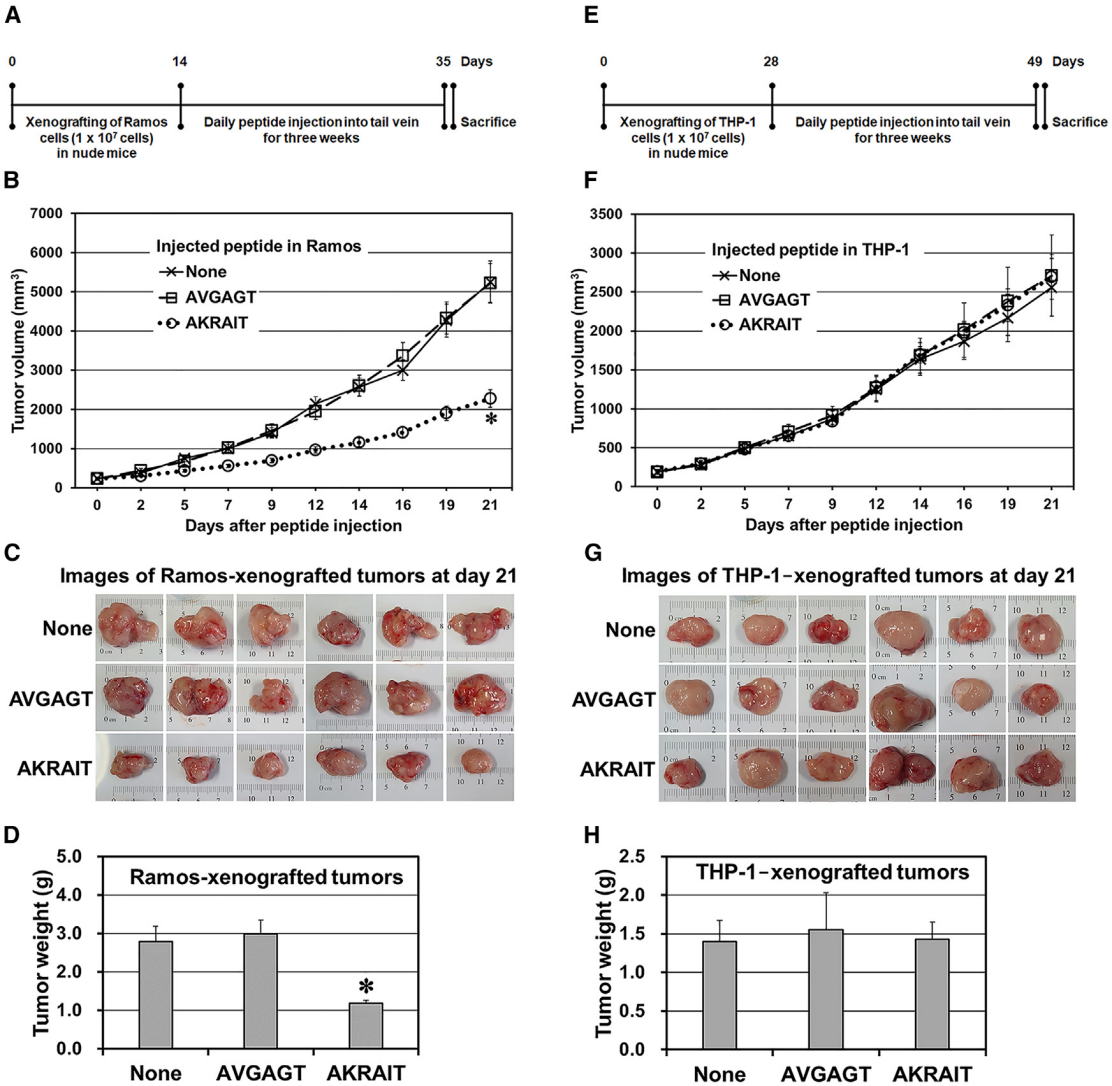
The volume and weight of Ramos-xenografted tumors in the athymic nude mice are reduced by the intravenous injection of the AKRAIT peptide (A and E) A schematic experimental time schedule is depicted for the xenografting of Ramos (A) or THP-1 (E) cells, as well as the administration of peptides. A set of six athymic nude mice for each kind of peptide was employed to xenograft Ramos or THP-1 cells. When the xenografted tumors were approximately 200 mm^3^ on average after the injection of 1 × 10^7^ cells as described in materials and methods, 1 mg peptide in 100 μL PBS per mouse was injected daily into the tail veins of xenografted nude mice. (B and F) The volumes (mm^3^) of Ramos xenografted (B) or THP-1 xenografted tumors (F) were measured *in situ* every 2 or 3 days for a period of 3 weeks to chase their growth trends. The chased trends of tumor volumes are presented in a line graph with error bars of SEM. ∗*p* ≤ 0.0005 (AKRAIT versus None in B). (C and G) The Ramos xenografted (C) or THP-1 xenografted (G) mice were sacrificed after 3 weeks of daily peptide injections to dissect out the xenografted tumors. The dissected-out xenografted tumors versus the injected peptides were photographed on a ruler to show the gross differences in volume and shape. (D and H) The final weights (g) of the dissected-out Ramos xenografted (D) or THP-1 xenografted (H) tumors were measured to be presented in a bar graph with error bars of SEM. ∗*p* ≤ 0.0017 (one-way ANOVA in D). The measured volumes and weights were statistically processed to obtain the SEMs and, if necessary, the *p* values.

The fact that the AKRAIT peptide had some intrinsic cell membrane permeability and thus inhibited the proliferation of Ramos cells by its direct addition to the cell culture system *in vitro* (described above) suggested that the peptide would also inhibit the growth of xenografted tumors of Ramos cells *in vivo* when the peptide by itself was injected intravenously into the xenografted nude mice without any further chemical modification and/or pharmaceutical formulation such as transfecting reagent, polymeric carrier, and/or liposome as a way to make it permeable through the cell membrane.^39^ As anticipated from the results of those preliminary experiments in the cell culture system *in vitro*, the AKRAIT peptide was found to have a significant anti-tumor efficacy in the Ramos-xenografted nude mice *in vivo* by just injecting daily the AKRAIT peptide by itself into their tail veins as compared to the PBS (None) and AVGAGT control peptide (Figures 7A–7D). Experimentally, the volumes of Ramos xenografted tumors were chased *in situ* by measuring them every 2–3 days for 3 weeks in parallel with the daily injection of 1 mg peptide in 100 μL PBS per mouse into the tail veins of xenografted athymic nude mice (Figure 7B). Finally, after 3 weeks of daily peptide injection, the xenografted mice were sacrificed by CO_2_ asphyxiation, and their xenografted tumors were dissected out to be photographed (Figure 7C) and to measure their final weights (Figure 7D). Surprisingly, the Ramos xenografted tumors were reduced about 60% in both the chased volumes *in situ* (Figure 7B) and final weights (Figure 7D) after 3 weeks of AKRAIT administration as compared to both PBS (None) and AVGAGT controls. However, the control experiments using the nude mice xenografted with the Crlz-1^−^ THP-1 cells showed no such anti-tumor efficacy (Figures 7E–7H).

## Discussion

To interfere with the intracellular protein-protein interaction, the potentially interfering peptide must pass through the cell membrane. However, the peptides cannot generally pass the membrane barrier when they are charged, polar, and/or large because the interior hydrocarbon chains of phospholipids within the cell membrane are hydrophobic as well as densely packed. In the cell culture system *in vitro*, this impermeability problem could be solved by using a transfecting reagent or electroporation, whereas it would be difficult to use such a method in the whole organism system *in vivo*. Recently, many approaches have been reported and reviewed to solve this problem of cell membrane impermeability in the cell culture system *in vitro* and eventually in the whole organism system *in vivo*.^36^^,^^37^^,^^38^^,^^39^ They might be grouped into two main categories, which are chemical modification and pharmaceutical formulation. To our great fortune, when the originally chosen AKRAIT peptide with an N-terminal label of FAM fluorophore was added directly to the cell culture media, which were RPMI-1640 supplemented with fetal bovine serum, l-glutamine, nonessential amino acids, penicillin/streptomycin, and 2-mercaptoethanol, but not sodium pyruvate, the fluorescent peptide was found to have some intrinsic cell membrane permeability by itself without any further chemical modification and/or pharmaceutical formulation (Figure 5A). The intrinsic cell membrane permeability of the FAM-labeled AKRAIT peptide has seemed against the general belief that the charged hydrophilic peptide molecules might be membrane impermeable. Nevertheless, this intrinsic permeability of positively charged AKRAIT peptide through the cell membrane could be explained by the model of complementary neutralizing electrostatic interactions between the surface negative charges of membrane phospholipids and the positively charged side chains of lysine and arginine residues of the AKRAIT peptide as postulated to explain the membrane permeability of the various CPPs, within which the positively charged amino acids such as lysine and arginine are often clustered and/or interspersed.^36^^,^^40^^,^^42^^,^^43^^,^^44^^,^^45^^,^^46^ In our hands, the model of complementary neutralizing electrostatic interactions employed to explain the intrinsic cell membrane permeability of the AKRAIT peptide might be supported by the observation that the intrinsic permeability was inhibited by the addition of sodium pyruvate as a growth-boosting ingredient to the cell culture media (Figure 5A). The inhibitory effect of sodium pyruvate on the intrinsic cell membrane permeability of the AKRAIT peptide by itself could be thought to be due to the neutralizing sequestration of the positively charged groups of the peptides by the negatively charged pyruvates, which might compete with the negatively charged phosphate groups of the membrane phospholipids.

To develop a more efficacious peptide derivative on the basis of the intrinsic membrane permeability of AKRAIT peptide by itself, the peptide has been modified further to improve its cell membrane permeability, its enzymatic stability, and its intracellular interfering capability against the relevant protein-protein interactions. Our tentative strategy to design various peptide derivatives of the KRAI motif has been to modify their N and/or C termini without any modification on the side chains of the KRAI motif to maintain their functional efficacies on the relevant protein-protein interactions. Because the solid-phase peptide synthesis starts after attaching the C-terminal carboxyl group to the solid phase, the N-terminal amino group has preferentially been utilized to modify the peptide. As suggested in recent reports and reviews,^43^^,^^44^^,^^45^^,^^46^ several preliminary peptide modifications while maintaining the KRAI motif have been tried to improve the peptide membrane permeability and/or its intracellular dominant-negative effects on the relevant protein-protein interactions. Those were N-terminal conjugation of consecutive arginine residues that is frequently employed to facilitate the passage of conjugated peptides through the phospholipid membrane by their electrostatic interactions,^40^ substitution of the C-terminal carboxyl group by hydroxyl group, or its amidation that abrogates the negative charge of the C-terminal carboxyl group, N-terminal FAM conjugation (or fluorescein isothiocyanate conjugation with a spacer of 6-amino hexanoic acid) that would add a hydrophobic fluorophore moiety, multimerization and/or cyclization of KRAI motifs that might increase its functional valency and peptidolytic stability. Thus far in our preliminary experiments, all these modifications have not much improved the cell membrane permeability and/or the proliferation inhibitory function in both the cell culture system *in vitro* and the xenografted nude mice of the whole organism system *in vivo*, although the multimerized and/or cyclized peptides have shown some promising improvement possibly due to their increased valency and/or stability. In addition, when the C-terminal carboxyl group was amidated or changed to the hydroxyl group to abrogate its negative charge, those modifications became worse in cell membrane permeability and/or functional efficacy. Other strategies to pursue for improving the anti-tumor efficacy of peptide derivatives of the KRAI motif could be a novel pharmaceutical formulation and/or chemical modifications, including stapling, peptide methylation, peptoid or d-amino acid substitution, and Fab or Fc conjugation. All of these could, individually or in combination, lead to better membrane permeability, better stability, and/or better delivery to the target tumor cells.

Finally, it should be noted that the KRAI peptide derivatives, together with their intrinsic cell membrane permeability, have two additional advantages of small molecular weight and water solubility due to a single stretch of short KRAI motif consisting of the charged amino acid residues, which could contribute to an easy and fast development of a much more efficient KRAI motif-based anti-BCL therapeutic peptide drug.

## Materials and methods

### Cell lines, their culture, and counting

Ramos (CRL-1596; American Type Culture Collection [ATCC]) is a human cell line of GC centroblast-derived BCL that was isolated from a Burkitt’s lymphoma patient, and Daudi (CCL-213; ATCC) is another human BCL cell line of Burkitt’s type. The control human cell lines of K562 (CCL-243; ATCC) and THP-1 (TIB-202; ATCC) wre originated from myelogenous and monocytic leukemia patients, respectively. NIH3T3 (CRL-1658; ATCC) is a mouse cell line of fibroblasts. The cells were cultured in RPMI-1640 media supplemented with fetal bovine serum (10%), l-glutamine (1×), nonessential amino acids (1×), penicillin/streptomycin (1×), 2-mercaptoethanol (50 μM), and with or without sodium pyruvate (1×) in a humidified 5% CO_2_ incubator at 37°C. Cells were counted on a hemocytometer (Neubauer; Marienfeld) after staining their aliquot with an equal volume of 0.4% trypan blue solution. All of the cell culture media and their supplements were purchased from Gibco (Thermo Fisher Scientific).

### Construction of Crlz-1 cDNA mammalian expression plasmid and derivation of its various truncation, deletion, and site-directed mutation constructs

mRNA was purified from PD36 mouse pre-B cells^3^ using FastTrack 2.0 (Invitrogen), and then a λ phage cDNA expression library was constructed using SuperScript Lambda System (GibcoBRL) and GigapackIII Gold Packaging Extract (StrateGene). A λ phage clone of wt full-length mouse *Crlz-1* cDNA was isolated by screening this PD36 cDNA expression library with a partial *Crlz-1* cDNA fragment generously provided by Dr. S.C. Bae.^1^ For the construction of wt full-length *Crlz-1* cDNA mammalian expression plasmid, the NcoI-NotI cDNA fragment taken from the plasmid of an isolated λ phage clone was filled using Klenow enzyme and subcloned into the EcoRV site of pCMV-Tag2A (Stratagene) in frame with the codons of FLAG tag peptide. Then, the *GFP*-fused *Crlz-1* mammalian expression plasmid was constructed by inserting the EcoRI-SalI fragment of the above pCMV-Tag2A-based *Crlz-1* expression plasmid into the corresponding sites of pEGFP-C1 (Clontech). Various *Crlz-1* truncations, deletions, and site-directed mutations with *GFP* at their 5′ sides were made by using various molecular biological techniques and a site-directed mutagenesis kit (Clontech) with care taken to maintain their coding frames. All these plasmid constructs were finally verified by sequencing.

### Transfection of *GFP*-*Crlz-1* plasmid constructs to localize their expressed proteins within cells

The wt or various mutant *Crlz-1* plasmid constructs with *GFP* on their 5′ sides as mentioned above were transfected into NIH3T3cells. The transfected cells were cultured on a coverslip within the wells of a 24-well plate for 2 days. After washing the cultured cells with PBS, they were fixed by 4% paraformaldehyde, washed with PBS, and then stained with Hoechst 33342 (100 μg/mL; Sigma) for 5 min. Finally, the stained cells, which were again washed three times with PBS, were mounted on a microscope glass slide to observe the cellular location of the expressed proteins using a fluorescence microscope.

### Peptide preparation and its transfection into cells with the use of a transfecting reagent

Stock solutions of various peptides, whose syntheses were custom ordered from GenScript via Koma Biotech in Korea, were first prepared by dissolving the lyophilized powder in PBS. Then, their aliquots were serially diluted with PBS to reach the final concentrations required for the experiments. The peptides were transfected into cells using Xfect Protein Transfection Reagent (Takara) basically following the experimental procedure as provided by the supplier. Briefly, the peptides in PBS (typically 0.4 μg) were mixed with Xfect buffer in a final volume of 100 μL, while 2 μL 1× Xfect reagent was mixed with the deionized water in a final volume of 100 μL. These two 100-μL solutions were mixed to be incubated at room temperature for 30 min. They were then added to 400 μL 2 × 10^5^ cells, which had previously been washed with PBS and resuspended in the serum-free warm RPMI-1640 media, and incubated to be transfected with a mild rotation at 37°C for 1 h. Finally, the 600 μL transfected cells were transferred to 3.4 mL complete media, which are RPMI-1640 supplemented with fetal bovine serum, l-glutamine, nonessential amino acids, penicillin/streptomycin, 2-mercaptoethanol, and sodium pyruvate. The 4-mL culture solution of transfected cells was divided in half to be duplicated for a 2-mL culture in each well of 6-well plate and then cultured in a humidified 5% CO_2_ incubator at 37°C to chase their cellular proliferation for a period of 3 days. To chase the effect of peptide transfection on the cellular proliferation, cells were counted daily on a hemocytometer by staining their aliquot with an equal volume of 0.4% trypan blue solution and finally harvested to analyze the effects of transfected peptide on various molecular events such as protein localization, transcription factor binding, gene expression, and rRNA production. For checking the cell membrane permeability of FAM-conjugated peptides in the Xfect transfections, the transfected cells were washed three times with warm PBS-T (0.01% Tween 20), fixed with 4% paraformaldehyde in PBS, and laid onto a poly-l-lysine-coated glass slide (PLL-01; Matsunami) with a cover glass using a mounting solution of DAPI (H-1200; VectaShield) to observe them using a confocal fluorescence microscope. For comparison, the experiment of KYA1797K treatment was performed in the same way as the peptide transfection.

### Western blot

The whole-cell protein extracts or their cytoplasmic and nuclear protein fractions of peptide-transfected cells, which had been prepared by NE-PER Nuclear and Cytoplasmic Extraction Reagents (Thermo Fisher), were electrophoresed on the SDS-PAGE gel using a protein electrophoresis system (Mini-PROTEAN; BioRad). The separated proteins in a resolving polyacrylamide gel were transferred to PVDF membrane (BPS0161; PALL) using the blotting apparatus of the same electrophoresis system (Bio-Rad). The blotted membranes were washed with TBS-T solution (137 mM NaCl, 3 mM KCl, 25 mM Tris, 0.05% Tween 20, pH 7.4), subsequently blocked with 5% nonfat dried skim milk in PBS-T, and probed for the relevant proteins by enhanced chemiluminescence reactions (sc-2048, Western Blotting Luminol Reagent; Santa Cruz Biotechnology) after treating them with the relevant primary and secondary antibodies in 1% skim milk TBS-T solution. The antibodies from Santa Cruz Biotechnology were anti-Sas10 (sc-56751), anti-PEBP2β (sc-56751), anti-Bcl-6 (sc-365618, sc-7388), and anti-β-actin (sc-130656), while the antibodies from Abcam were anti-UTP-3 (ab60063), anti-CBFβ (ab33516), anti-β-actin (ab8227), anti-Lamin-B1 (ab16048), and horseradish peroxidase-conjugated anti-rabbit IgG (ab6721, ab205718).

### ChIP

ChIP was performed essentially as described previously.^3^^,^^4^ The antibodies used in our ChIP experiments were normal rabbit IgG (sc-2027; Santa Cruz Biotechnology) and anti-CBFβ (ab33516; Abcam). The primer sequences for the PCR of the ChIP experiments are given with their annealing temperatures and amplicon DNA sizes in Table 1.

**Table 1:** Sequences of primer pairs of human genes

Name	Forward primer	Reverse primer	Annealing temperature, °C	DNA size, bp
CBFβ	tcg tgc ccg acc aga gaa gc	tca gaa tca tgg gag cct tc	59	300
Crlz-1	atg ttg cga aag gaa tca cc	gac agg atg tcc atg tgc tg	57	240
Bcl-6	cat gca gag atg tgc ctc cac a	tca gag aag cgg cag tca cac t	60	133
Cyclin D1	ccg tcc atg cgg aag atc	gaa gac ctc ctc ctc gca ct	58.5	75
Cyclin D2	tac ttc aag tgc gtg cag aag gac	tcc cac act tcc agt tgc gat cat	60.5	338
Cyclin D3	cga gcc tcc tac ttc cag tg	gga cag gta gcg atc cag gt	59.5	150
Blimp-1	cag ttc cta aga acg cca aca gg	gtg ctg gat tca cat agc gca tc	59	123
IRF-4	atg ctt tgg aga gga gtt tc	ctg gat tgc tga tgt gtt c	53.5	158
IgJ	gga gtc ctg gcg gtt ttt a	agg cat ctg ggg tta agg ct	56.5	442
β-Actin	cac cat tgg caa tga gcg gtt c	agg tct ttg cgg atg tcc acg t	56	135
Bcl-6-proximal (ChIP)	ggg gct ggg att gtt aca tat ggc c	gct ctt tcc aac tag aat att agg c	60.5	372
Bcl-6-distal (ChIP)	agg gac aga cac ttt aac gca	ttg gac cag aag cag ctt tca	56.5	435

### Extraction of total RNA, reverse transcription-polymerase chain reaction, and electrophoresis

Total RNA was extracted from the peptide-transfected cells using Trizol reagent (Invitrogen) following the procedure as provided by the supplier and then treated by RNase-free DNase I (Qiagen) to remove any possible DNA contamination. Total RNA as extracted, precipitated, rinsed, and dried to a just-damp state by the usual methods was dissolved in 50 μL RNase-free water to obtain a working concentration range of 0.5–1 μg/μL. One microgram of this final dissolved RNA stock was reverse transcribed at 45°C for 1 h using RT PreMix (iNtRON, Korea), with an adjustment of total reaction volume to 20 μL by adding RNase-free water. After stopping the cDNA synthesis reaction by heating at 95°C for 5 min, its volume was then adjusted with an addition of RNase-free water to the final volume of 100 μL, 5 μL of which was taken and added to a PCR premix (K-2016; Bioneer, Korea), with 1 μL of a pair of gene-specific primers (10 pmol/μL for both forward and reverse primers) in a final water-adjusted volume of 20 μL to amplify the relevant cDNA by PCR. The PCR conditions with various pairs of gene-specific primers and their sizes of amplified DNA are presented in Table 1. The levels of 18S and 28S rRNAs among the total RNA, which were extracted from the same numbers of peptide-transfected cells, were compared by staining with EtBr after separating them on the same 1% agarose gel.

### Direct addition of peptide by itself to cells in the culture media without using a transfecting reagent

For a 2-mL culture of cells in each well of a 6-well plate, 7 μL peptide solution in a concentration of 10 μg/μL in PBS was added directly to the culture media, which were RPMI-1640 supplemented with fetal bovine serum (10%), l-glutamine (1×), nonessential amino acids (1×), penicillin/streptomycin (1×), and 2-mercaptoethanol (50 μM), but not sodium pyruvate. The intrinsic cell membrane permeability of peptide with its N-terminally conjugated FAM fluorophore was checked by observing the peptide-treated cells in a confocal fluorescence microscope (Zeiss) after mounting the washed cells on a microscope slide with a cover glass. It was important that the treated cells be washed well, for example, five times with warm PBS-T (0.01% Tween 20) to remove any nonspecifically adsorbed FAM fluorophore-conjugated peptides. Various custom-ordered peptides with or without the conjugated FAM fluorophore to be used for this direct addition to the cells in culture media as well as for their intravenous injection to the xenografted nude mice as described below were prepared as mentioned above. Among them, AKRAIT is the NLS peptide of Crlz-1 protein, whereas AVGAGT (hydrophobic), ADEAIT (negatively charged), and TIARKA (oppositely sequenced) are three differently designed control peptides to verify the critical values of lysine, arginine, and isoleucine residues of KRAI motif and its sequence direction.

### Xenografting of cells in the athymic nude mice and intravenous injection of peptides

The animal experiments using athymic nude mice (5-week-old female), which were purchased from Koatech, were performed with the approval (KHGASP-24-195) of the Kyung Hee University Institutional Animal Care and Use Committee and according to its guidelines.

To prepare cells for xenografting 10^7^ cells per mouse, multiples of logarithmically growing 10^7^ cells were pelleted, resuspended in the multiples of 200 μL cold PBS, and then mixed homogenously with the corresponding multiples of 200 μL cold Matrigel (Corning), taking a great deal of care to avoid foam. The suspension of 10^7^ cells prepared as above was injected just underneath the skin on the right back flank of each athymic nude mouse (5-week-old female; Koatech), also taking care to avoid any deep puncture. When the xenografted tumors had been roughly 200 mm^3^ after the injection of cells, 1 mg peptide in 100 μL PBS per mouse was started to be injected daily into the tail veins of xenografted nude mice. The volume of tumor *in situ* was calculated using the equation (long diameter × short diameter^2^)/2 by measuring its long and short diameters using a caliper (CD-15APX; Mitutoyo). The xenografted nude mice were sacrificed by CO_2_ asphyxiation after a certain period of daily peptide injection, and the xenografted tumors were dissected out to be photographed, as well as to measure their final weights.

### Statistical analysis

Error bars in the graphs represent the standard error of the mean (SEM). The paired two-tailed Student *t* test was usually employed to calculate the *p* value, while one-way ANOVA was used in Figure 7D. *p* values less than 0.05 were considered statistically significant.

## Data and code availability

The data and materials for the published results are available upon request from the corresponding authors.

## Acknowledgments

This work was supported by the 10.13039/501100003725National Research Foundation of Korea (NRF) grant funded by the Korean government (10.13039/501100014188Ministry of Science and ICT) (2022R1F1A1064199).

## Author contributions

J.H.P. and S.Y.C. performed almost all the experiments. S.-K.P. started the founding experiments of basic concepts. J.L. supplied ideas and advice on the experimental techniques along with troubleshooting. C.J.K. supervised all the experimental work with the secured research grant, wrote the manuscript, and configured and/or prepared the figures. All authors have read the manuscript and confirmed their contributions to it.

## Declaration of interests

C.J.K., S.Y.C., and J.H.P., along with Kyung Hee University, filed Korean patent application 10-2024-0039616 on March 22, 2024 (Nuclear Localization Signal Peptide and Uses Thereof).
